# Chemical Warfare Through the Ages: A Systematic Review From Antiquity to the Present

**DOI:** 10.1155/jt/7363632

**Published:** 2025-11-20

**Authors:** Damian Alexander Honeyman, David James Heslop, Samsung Lim, Chandini Raina MacIntyre

**Affiliations:** ^1^Biosecurity Research Program, The Kirby Institute, The University of New South Wales, Kensington, New South Wales, Australia; ^2^School of Population Health, The University of New South Wales, Kensington, New South Wales, Australia; ^3^School of Civil and Environmental Engineering, The University of New South Wales, Kensington, New South Wales, Australia; ^4^College of Public Service and Community Solutions, and College of Health Solutions, Arizona State University, Tempe, Arizona, USA

**Keywords:** chemical agents, chemical warfare, chemical weapons, weapons of mass destruction history

## Abstract

Chemical warfare means the use of chemical agents that have direct toxic effects on animals, plants and humans, as weapons. The first documented use of a chemical agent for warfare purposes occurred in ancient times around 10,000 BCE in South Africa when weapons were dipped in chemicals and then used to attack and defend from enemies. However, much of the evidence lacks detail to provide thorough accounts of such events. Nevertheless, we aimed to systematically gather the most comprehensive account of all publicly known incidents involving chemical weapons throughout history. We identified 121 instances of chemical weapon use between 10,000 BCE and October 2023 spanning 49 countries and causing at minimum 2,110,360 injuries and 2,930,769 deaths. Across the 121 incidents, at least 165 chemical agents were used. Of the known chemical agents, the top three were sulphur mustard (*n* = 16, 12.1%), hydrogen cyanide (*n* = 12, 7.3%) and chlorine gas (*n* = 11, 6.7%). Of the known chemical classes, the top three used were vesicants (blistering agents) (*n* = 31, 18.8%), choking (pulmonary) agents (*n* = 18, 10.9%) and nerve agents (*n* = 18, 10.9%). If a chemical agent was not reported, the chemical class was reported as unknown (*n* = 35, 21.2%). A small number of chemical weapons were used that fell outside of the main categories of agents (*n* = 20, 12.1%). Chemical weapons remain a serious concern locally and globally, and there are few data on the global epidemiology of such incidents. Prevention, early detection and rapid response are key and can be enabled by global surveillance for chemical incidents.

## 1. Introduction

Throughout history and as early as 10,000 BCE, humans as state and nonstate actors have identified and used naturally occurring poisons, toxins, noxious fumes, incapacitants and incendiaries as chemical weapons to achieve victory over opponents in chemical warfare [1].

Chemical warfare is defined as the use of the toxic properties of chemical substances with the aim to kill, injure or incapacitate an enemy during warfare [2]. These chemical substances are commonly referred to as chemical warfare agents (CWAs). They are widely known for being capable of causing immense physical and psychological trauma, with their impact causing gruesome effects on the human body while often being invisible but highly destructive [3]. Their effects can be immediate, acute and/or chronic longer term; however, their action depends on the chemical dose, duration and route of exposure (e.g., dermal/eye absorption, ingestion and inhalation) [4]. They can be dispersed as a gas, liquid or aerosol or as agents that can be absorbed into particles to become a powder [5]. Such CWAs are regarded as among the most brutal weapons of mass destruction (WMD) known to humankind [5]. They can cause mass casualties and injuries, destabilise governments, create conditions that exacerbate home-grown violence and promote terrorism while also adversely affecting ecosystems and the environment [6]. They can be classified by their primary effect as either lethal, incapacitating or harassing [7]. Each CWA possesses different characteristics while being grouped into various classes with pronounced physiochemical, physiological and chemical property differentiators [8]. The main CWA classes are nerve agents, vesicants (blistering agents), blood agents (cyanogenic agents), choking (pulmonary) agents, riot-control agents (tear gases), psychomimetic agents and toxins [5] and, more recently, a new class of binary and nontraditional agents [7]. Based on their volatility, they can be classified as persistent or nonpersistent agents. The more volatile an agent is, the quicker it will evaporate and be dispersed and may only be a hazard for a defined period of time. By contrast, persistent agents cause long-term contamination and can result in the tactical effect of area denial or obstacle through contamination of large areas of ground and foreclosing avenues of movement and supply [9].

The Chemical Weapons Convention (CWC) prohibits the production, development and use of CWAs under the CWC, which came into force in 1997. Signatory countries were required to destroy chemical weapon stockpiles within a 10-year period. However, of the 193 countries that signed the CWC, 27 are yet to ratify [10]. The use of CWAs has not been eliminated by the CWC, complicated by the ease in which the manufacture of certain precursor chemicals has a dual use for legitimate economic and industrial purposes. Additionally, new agents continue to be developed and fielded by state and nonstate actors with multiple examples of offensive use against individuals and populations in the past 10 years. The scope of potential threats is extremely broad with the U.S. Centres for Disease Control (CDC) listing 73 separate chemical threats, including pesticides; explosive nitro compounds; flammable industrial gases and liquids; poisonous industrial gases, liquids and solids; corrosive industrial acids and bases; dioxins, furans and polychlorinated biphenyls (PCBs); toxic alcohols; and long-acting anticoagulants and receptor ligands [11]. Therefore, while many chemical agents were initially developed for commercial purposes, many can also be weaponised for offensive use with relative ease [7]. The diversity of possible chemical threats presents a major challenge to nonproliferation and chemical defence efforts around the world. One important approach in understanding which chemical hazards present the most significant threat, and thus inform rational chemical defence policy, preparedness and response, is to examine the history of instances of CWA use. Comprehensive lists of CWA events and their impact across historical timeframes are not readily available, with most recent information often securitised and inaccessible to scientific enquiry. Thus, the purpose of this study was to conduct a systematic review of historical chemical warfare events.

## 2. Methods

In this study, we systematically reviewed the impact and features of historical chemical warfare events, and how these have evolved over time.

### 2.1. Study Protocol

This systematic review was performed in accordance with the Preferred Reporting Items for Systematic Reviews and Meta-analysis (PRISMA) guidelines [12]. The protocol for this review was recorded on the International Platform of Registered Systematic Reviews and Meta-analysis Protocols (INPLASY) with the registration ID INPLASY202430016.

### 2.2. Search Strategy

A preliminary literature review was conducted through Google Scholar with general topic terms, such as ‘chemical warfare', ‘weapon', ‘agent' and ‘history', to identify scope and previous work in this area by other researchers. We identified references for this review by searching PubMed, Web of Science and Scopus for articles published with an open date range and up to 7 March 2024, using the terms ‘history', ‘chemical' and ‘warfare' (Appendix 1). A second Google search was undertaken to identify chemical warfare events in the grey literature. Expert consultation was undertaken with a specialist in the CBRNE field (DJH), to locate published books in the field to be added for review. We reviewed these articles and books with relevant references that were cited. We included articles and books published in English in the search. Journalistic and open-source intelligence (OSINT) were not systematically searched, as our inclusion criteria focused on verifiable peer-reviewed and grey literature sources.

### 2.3. Inclusion and Exclusion Criteria

Reports were included if they described incidents of chemical warfare affecting humans throughout history. Only chemical warfare events where evidence supported that an event took place were considered. All publicly known chemical warfare incidents were included from single-target assassinations to mass casualty events. We also included chemical warfare events where the chemical agent class and chemical agent or weapon were unknown, but evidence supported (on balance) the offensive use of a chemical agent. Our study also covered chemical agent use whereby the exact event descriptives were not stated but enough relevant information to state it had occurred was available in any given report. Additionally, chemical weapon use at events was included if exact injury and death figures were not stated, due to the underlying assumption that not all injuries and deaths are recorded. Reports were excluded if (a) they stated there was threatened use of or unconfirmed use of chemical weapons; (b) the reports were not available in full text; and (c) the reports were not published in the English language.

### 2.4. Data Extraction

One reviewer (DAH) conducted data extraction using a standardised spreadsheet (Table 1). A second author (DJH) cross-checked the information to verify correctness. Discrepancies between reviewers were resolved through consensus, and the data were then validated by domain experts (DJH and CRM). We extracted information and compiled it into a summary table by date range from the first chemical warfare event. For the summary table variables, we recorded the following data to ascertain the characteristics of events: year of event, chemical agent class, chemical agent or weapon used, chemical origin, geospatial coordinates of event, geographical location, event descriptives, mode of agent delivery and injuries and deaths.

### 2.5. Quality and Risk-of-Bias Assessment

A formal quality and risk-of-bias assessment was not feasible given the heterogeneity of sources and the historical nature of the data. However, the veracity and accuracy of historical reports were carefully evaluated using an adjudication process. Each event was independently reviewed, and where there were conflicting accounts or controversy regarding attribution (e.g., offensive versus nonoffensive use), decisions were made through consensus among subject matter experts (DJH and CRM) and the chief investigator (DAH). Priority was given to peer-reviewed publications and corroborated reports over single or anecdotal accounts. To increase transparency, we adapted elements from the ROBIS and PRISMA frameworks by considering three domains: (i) reliability of the primary source (peer-reviewed, official or anecdotal), (ii) consistency across independent accounts and (iii) toxicological plausibility of the described events. Events deemed unverifiable or lacking corroboration were excluded. While this approach strengthens confidence in the dataset, residual uncertainty remains inherent in historical sources, which we explicitly acknowledge as a limitation.

### 2.6. Analysis

We analysed the summary table (Table 1) using STATA/BE 18.0 for Mac and Microsoft Excel. We performed cross-tabulations on variable data for chemical agent class, chemical agent, geographical location and injury and death numbers to generate summary information for chemical weapon use. We generated a summary data figure using ArcGIS Pro v.3.1.

**Figure 1:**
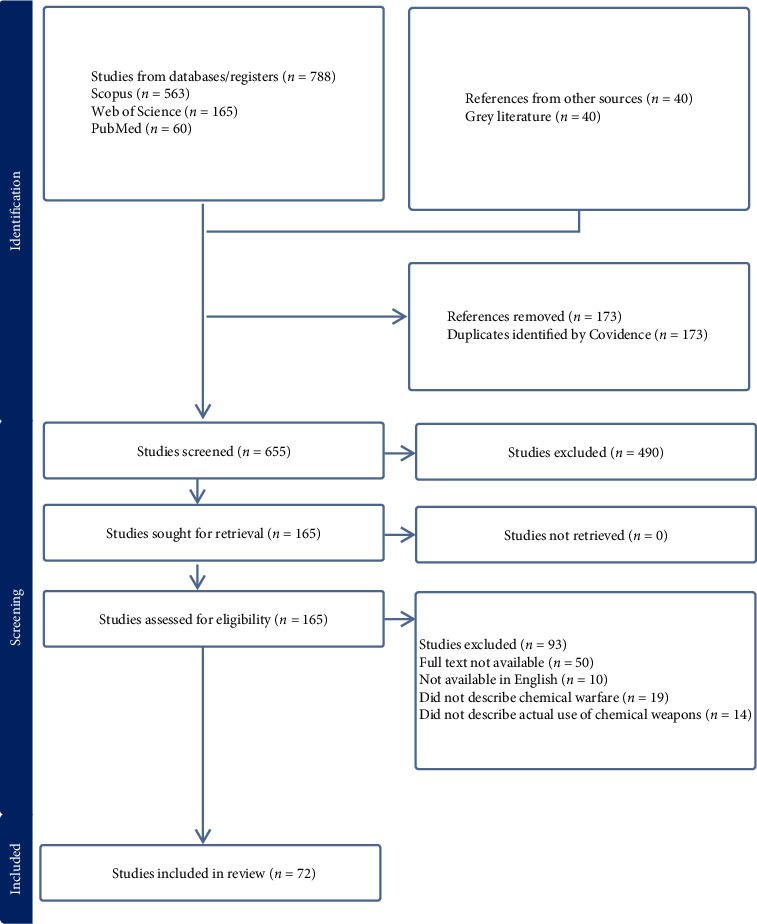
Flow diagram of included reports.

In total, 788 reports were identified by the search strategy. After eliminating duplicate titles and completing a review of article titles, abstracts and full texts, 72 reports were included in the analysis (Figure 1).

## 3. Results

### 3.1. Summary and Demographics

**Figure 2:**
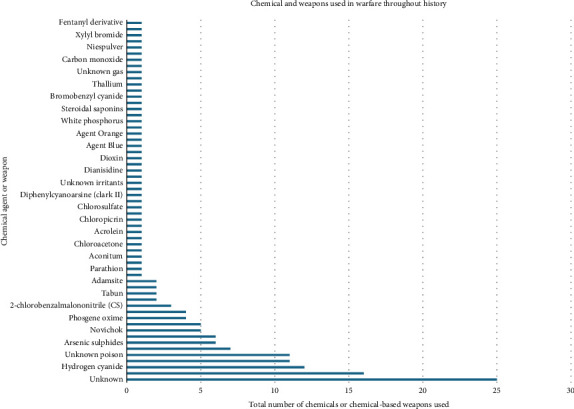
Chemicals and weapons used throughout history for chemical warfare. This is an original figure created by the authors of the manuscript.

In this study, we identified 121 instances of chemical warfare dating back to 10,000 BCE (Table 1) where at least 2,110,360 injuries and 2,930,769 deaths were recorded. We identified 62 different chemical agents or weapons that were used for chemical warfare (Figure 2). Of the 62 identified agents, 44 (71.0%) were synthetic or industrial in origin, six (9.7%) were plant-derived (e.g., aconitine, belladonna alkaloids and *Gelsemium elegans*), three (3.2%) were microbial/fungal toxins (ricin, ergot and alkaloids) and four (6.5%) were mineral or elemental agents (arsenic sulphides, sulphur, thallium and white phosphorus). The remaining five agents (8.1%) were classified as of unknown origin.  Table 2 summarises the toxicological properties of representative CWAs. While chemical structures are not reproduced here, they are available in published work, such as ‘Research Progress in the Degradation of Chemical Warfare Agent Simulants Using Metal–Organic Frameworks' [13].

**Figure 3:**
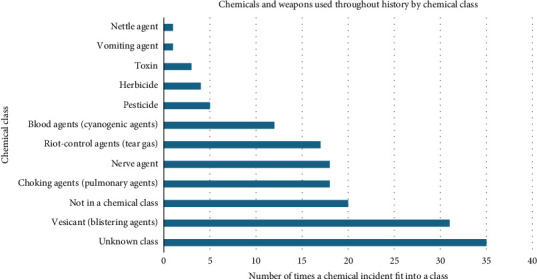
Chemical agents and weapons used throughout history by chemical class. This is an original figure created by the authors of the manuscript.

**Figure 4:**
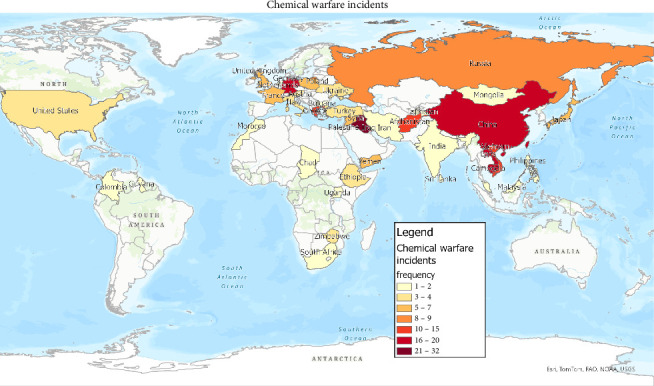
Global map of countries where chemical warfare incidents have taken place throughout history. This is an original figure created by the authors of the manuscript.

**Figure 5:**
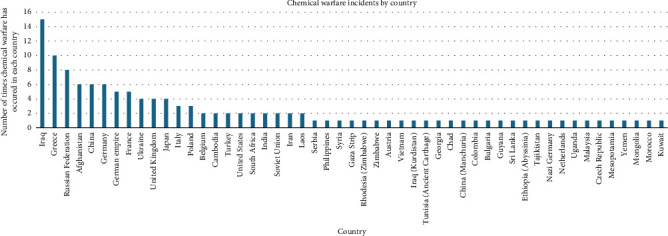
Total number of countries where chemical warfare incidents have taken place throughout history. This is an original figure created by the authors of the manuscript.

Across the 121 events, chemical agents were used 165 times. Of the known chemical agents, the top three were sulphur mustard (*n* = 16, 12.1%), hydrogen cyanide (*n* = 12, 7.3%) and chlorine gas (*n* = 11, 6.7%). Of the known chemical classes, the top three used were vesicants (blistering agents) (*n* = 31, 18.8%), choking (pulmonary) agents (*n* = 18, 10.9%) and nerve agents (*n* = 18, 10.9%) (Figure 3). If a chemical agent was not reported, the chemical class was reported as unknown (*n* = 35, 21.2%), and if the chemical was stated but was not regarded as one of the seven classes, it was reported as ‘not in chemical class' (*n* = 20, 12.1%). The chemical warfare events spanned 40 countries; the top three were Iraq (*n* = 16, 13.9%), Germany (*n* = 12, 10.4%) and Greece (*n* = 10, 8.7%) (Figures 4 and 5).

### 3.2. Early Use of Chemical Warfare: Ancient Times

Chemical agents were recognised for their tactical potential as early as 10,000 BCE, with archaeological evidence from southern Africa suggesting the use of poisoned arrowheads tipped with animal venoms (scorpion and snake venom) and plant extracts [1]. Ancient armies across India, China and the Mediterranean similarly employed toxic smokes and water contamination as weapons [14–16]. Examples include arsenic-laced ‘soul-hunting fog' in China [14, 17], the poisoning of wells with hellebore roots during the Siege of Kirrha in Greece [14, 18, 19] and the Assyrian use of ergot alkaloids to taint enemy water supplies [1]. Other accounts describe narcotic agents, such as mandrake root, being used to incapacitate opponents [14, 19].

While the accuracy of these records is often uncertain, they reveal recurring patterns of early chemical warfare, including the reliance on natural toxins (such as plants, venoms and alkaloids), the strategic targeting of food and water supplies, and the use of smoke to disorient or incapacitate enemies. These patterns demonstrate an early recognition of chemical toxicity and its utility in asymmetric or siege warfare.

### 3.3. The Common Era to World War I

From the first century CE through the late mediaeval period, chemical weapons were increasingly documented in both European and Asian warfare. Roman accounts describe the use of cyanogenic compounds from cherry laurel, while mediaeval Chinese and Mongol armies developed incendiary compounds from cherry laurel, nitre (saltpetre), petroleum and resins to generate suffocating smokes and firebombs [1]. The most iconic innovation of this period was ‘Greek Fire', a petroleum-based incendiary that ignited on contact with water and was developed by the Byzantine navy for centuries [1, 20, 21]. Arsenic sulphides, aconite alkaloids and other plant or mineral toxins were also weaponised for poisoning wells, coating arrows or producing choking fumes. For example, historical analyses [1, 14] describe the use of arsenic-based compounds in early siege warfare, while additional studies [22–24] provide corroborating accounts of toxic smokes and fume-producing mixtures used across Asia and the Mediterranean.

By the early modern period, the use of chemical irritants and incendiaries became increasingly engineered, as illustrated by sulphur- or resin-based ‘stink bombs' and arsenic-laced projectiles. Although many accounts exaggerate their effectiveness, these examples reflect a thematic shift from opportunistic natural poisons to more deliberate formulations, laying the groundwork for industrial-scale synthesis in the twentieth century.

### 3.4. Warfare From World War I

The First World War—often remembered as ‘The Chemist's War'—marked the beginning of modern, industrialised chemical warfare. For the first time, laboratory-synthesised toxicants, such as chlorine, phosgene, mustard gas and chloropicrin, were deployed on a massive scale. At the Second Battle of Ypres in 1915, German forces released chlorine gas, causing thousands of pulmonary injuries and demonstrating the effectiveness of CWAs on the battlefield. First-hand military medical reports of these chlorine attacks have been analysed and documented [1, 19]. Tactical deployment and dispersal methods were analysed in subsequent studies [22], while toxicological assessments [25–27] quantified the extent of pulmonary injury and mortality among the exposed soldiers. Chlorine acted as a corrosive pulmonary agent, hydrolysing to hydrochloric acid in the airways and inducing acute lung injury and hypoxia [28]. This was soon followed by large-scale use of phosgene and diphosgene, pulmonary agents that produced delayed respiratory failure and significantly increased fatality rates. Phosgene-induced pulmonary oedema accounted for substantial mortality, as reported in early wartime analyses [19, 21, 22], a finding later echoed by later epidemiological studies [27, 29, 30] that confirmed fatality rates exceeding those of chlorine. Phosgene's toxicology is notable for its latent period: victims often appeared stable initially, only to develop fatal pulmonary oedema hours later [31].

In 1916, French forces introduced hydrogen cyanide, the first battlefield use of a blood agent, followed by chlorine the same year. Contemporary military accounts and toxicological analyses [19, 32, 33] describe the deployment and physiological effects of these agents, highlighting their limited tactical success but significant psychological impact on opposing forces. In 1917, the German Empire escalated further with the deployment of sulphur mustard (‘mustard gas'), which caused blistering skin lesions, ocular damage and chronic respiratory sequelae due to its DNA-alkylating properties [24, 33, 34]. Mustard exposure not only produced acute vesication but has also been linked to long-term carcinogenesis, pulmonary fibrosis and reproductive toxicity in survivors [35]. Mustard gas became the most notorious agent of the war, responsible for long-term morbidity among survivors, and still regarded as one of the most consequential CWAs in history [32, 33].

Other agents were also tested. Tear gases (ethyl bromoacetate and chloroacetone), vomiting agents, such as diphenylchlorarsine (Clark I), and a wide range of irritants, including acrolein, bromoacetone and bromobenzyl cyanide, were also used with varying effectiveness. Historical and toxicological accounts [20, 27, 36] describe their experimental use, noting that although these agents caused fewer casualties than chlorine, phosgene or mustard gas, they provided valuable insights into chemical dispersal and battlefield delivery methods. Blistering arsenical agents, such as phenyldichloroarsine (Yperite) and methyldichloroarsine, were also introduced in 1917–1918 [1, 19, 26]. While many of these caused fewer casualties than the ‘big three' (chlorine, phosgene and mustard), their testing reflected rapid chemical innovation driven by the conflict.

By the end of the war, an estimated 1.3 million soldiers had been injured and 90,000 killed by CWAs [30, 34]. Beyond their immediate toll, these events set a precedent for the industrial production and systematic deployment of CWAs and established their toxicological hallmarks to continue chemical warfare: pulmonary agents causing acute lung injuries, vesicants inducing chronic carcinogenesis and fibrosis, and systemic poisons with widespread physiological effects.

### 3.5. The Interwar Period

After the First World War, international efforts to curb chemical warfare led to the signing of the 1925 Geneva Protocol, which prohibited the use of asphyxiating, poisonous gases and other bacteriological methods in warfare. Earlier precedents included the 1675 Strasbourg Agreement and the Hague Conventions of 1899 and 1907 [37]. Despite these attempts at regulation, enforcement was weak, and CWAs continued to be deployed in conflicts throughout the interwar years.

Small-scale uses occurred during the Russian Civil War, when British forces employed diphenylchlorarsine with limited effect [38], and the Red Army later used chlorine shells against peasant rebels in Tambov in 1921 [39]. More consequentially, the Rif War (1921–1926) in Spanish Morocco marked the first systematic postwar campaign of chemical warfare, with Spanish forces repeatedly using gas shells and mustard bombs against Riffian fighters [1, 20, 40].

The 1930s witnessed a resurgence of mustard gas use by both the Soviet military against Chinese troops in Xinjiang (1934–1937) and Fascist Italy during its invasion of Abyssinia (now modern-day Ethiopia) (1935–1936). Historical and military analyses [19, 23, 38] document these campaigns in detail. Italian forces escalated deployment by dropping mustard bombs, spraying from aircraft and applying powdered forms on the ground, contributing to an estimated 15,000 Ethiopian casualties [1, 41]. These events underscored the potential of aerial delivery methods and foreshadowed large-scale chemical strikes in later conflicts.

The Second Sino-Japanese War (1937–1945) further demonstrated the persistence of CWAs, as imperial Japan employed mustard gas, lewisite, phosgene oxime, hydrogen cyanide and tear gases in a variety of munitions, causing at least 72,000 injuries and 10,000 deaths. Historical and military analyses [20, 23, 24] detail these operations, highlighting their extensive human toll and the diversification of chemical arsenals during this period. By contrast, reports of Polish use of mustard gas in Jaslo in 1939 remain poorly substantiated [38].

The interwar period highlights the fragility of international agreements in the face of strategic and colonial ambitions. Despite legal prohibitions, chemical weapons were repeatedly used to suppress insurgencies, intimidate civilian populations and experiment with new delivery systems, such as aerial spraying, reinforcing their role as both tactical and terror-inducing weapons.

### 3.6. Chemical Warfare From World War II

Before World War II, there was widespread expectation that chemical weapons would again play a decisive battlefield role. However, large-scale use in combat was largely avoided, possibly due to the deterrent effect of mutual stockpiling and fear of retaliation. Instead, chemical agents were deployed primarily in the context of genocide. Nazi Germany weaponised Zyklon B, a cyanide-releasing pesticide, as part of its extermination programme, killing millions of the Jewish population, Romani communities, people with disabilities and other groups labelled ‘undesirable'. Historical and toxicological accounts [21, 22, 24] describe the development, large-scale distribution and use of Zyklon B within extermination camps, where it became the most notorious chemical agent of the twentieth century [1]. The toxicological mechanism, rapid cellular asphyxiation due to inhibition of cytochrome oxidase, made it both efficient and devastating in enclosed environments.

Although battlefield deployment of CWAs did not occur on the same scale as in World War I, the Holocaust highlighted their potential for mass extermination and underscored the fragility of humanitarian protections. In a lesser known episode, in April 1946, a group of Holocaust survivors known as ‘Nakam' (‘revenge' in Hebrew) allegedly poisoned SS officers and high-level Nazi staff in Stalag 13 with arsenic in retaliation for wartime atrocities [9].

The Second World War demonstrated that the absence of large-scale battlefield use did not equate to the end of chemical warfare. Instead, CWAs were repurposed for state-led genocide, cementing their association not only with military conflict but also with crimes against humanity.

### 3.7. The Post–World War II Period and the Cold War

The postwar period saw rapid advances in chemical agent development, alongside repeated battlefield use despite international prohibitions. In 1952, the United Kingdom developed VX at Porton Down, the first of a new class of highly potent organophosphorus nerve agents [42]. The formula was later transferred to the United States in exchange for thermonuclear weapons information, accelerating the chemical arms race [43]. The Soviet Union subsequently synthesised the even more powerful Novichok series, underscoring the intensification of Cold War toxicology research. VX and Novichok act through irreversible inhibition of acetylcholinesterase, leading to cholinergic crisis characterised by seizures, paralysis and respiratory failure. Survivors often suffer persistent neurocognitive and neuromuscular impairments [44].

On the battlefield, CWAs were deployed in multiple regional conflicts. During the Vietnam War (1962–1975), the United States and South Vietnam used riot-control agents, such as 2-chlorobenzalmalononitrile (CS), a-chloroacetophenone (Adamsite), while also conducting large-scale herbicidal campaigns with ‘Agent Orange' and related mixtures (n-butyl 2,4-dichlorophenoxyacetate [2,4-D], n-butyl 2,4,5-trichlorophenoxyacetate [2,4,5-T], iso-butyl trichlorophenoxyacetate, cacodylic acid or picloram). Archival United States military reports [9] describe extensive deployment in defoliation campaigns, while contemporaneous studies [20, 21, 40] identified toxicological persistence of dioxin contaminants; epidemiological reviews [26, 45] later linked these exposures to multigenerational health effects.

Although primarily intended for defoliation, these chemicals were later shown to exert systemic toxicities including endocrine disruption, teratogenesis and carcinogenesis, with health effects persisting across generations of exposed populations [46]. Exact casualty estimates remain uncertain, but the scale of exposure was unprecedented.

Elsewhere, Egyptian forces reportedly used mustard gas, tear gas and possibly nerve agents during the Yemen Civil War (1963–1967), causing several hundred injuries and deaths. Documentary records from the International Red Cross [9] and eyewitness accounts [19, 20] reported widespread civilian suffering, while military analyses [24, 26, 40] and a retrospective toxicology study [47] confirmed vesicant-related respiratory and dermal injuries consistent with mustard gas exposure.

In Rhodesia (1976–1978), parathion and thallium were allegedly used against insurgent forces (Zimbabwe African National Liberation Army (ZANLA)), delivered through poisoned wells and contaminated clothing [9, 48]. North Vietnamese and Lao troops used lethal and incapacitating chemicals against the Hmong resistance between 1978 and 1983 [9, 49]. Soviet forces in Afghanistan (1979–1989) were accused of deploying phosgene oxime, nerve agents and riot-control gases [1].

The most consequential CWA campaign of the Cold War occurred during the Iran–Iraq War (1980–1988). Iraq employed sulphur mustard, tabun and sarin against both Iranian soldiers and civilians, marking the first battlefield use of the latter two agents since their synthesis in the 1930s. Primary accounts [18–20] describe the rediscovery and production of tabun and sarin, while operational analyses [27, 29, 32] outline their field deployment. More recent reviews [50–52] confirm the Iran–Iraq War as the first verified battlefield deployment of these nerve agents.

These attacks caused mass casualties, an estimated 30,000 deaths and tens of thousands of long-term injuries, including pulmonary fibrosis, cancers and reproductive impairment [9]. In 1988, the Halabja massacre, in which sulphur mustard and sarin killed approximately 5000 Kurdish civilians and injured 10,000 more, remains one of the most notorious chemical attacks in history [53–55]. Smaller scale use was also documented in Libya's 1987 attacks on Chad [1].

The Cold War era reflected two defining trends: (i) the technological escalation from vesicants to advanced nerve agents (VX, Novichok) and (ii) the persistence of CWAs in regional conflicts, often targeting civilians or insurgent groups. Together, these patterns underscored both the enduring military appeal of chemical weapons and their catastrophic long-term toxicological impact.

### 3.8. Persian Gulf War

During the Persian Gulf War, Iraq's invasion of Kuwait led to a United States–led coalition offensive in early 1991. Although large-scale battlefield use of CWAs did not occur, concern entered Iraq's substantial stockpiles of nerve agents, such as sarin and soman. Coalition bombing of suspected weapons storage facilities raised the possibility of low-level exposures among deployed personnel. In the aftermath, many veterans developed a constellation of chronic symptoms from chronic impairment to musculoskeletal pain, and gastrointestinal disturbance, collectively termed ‘Gulf War Illness or Gulf War Syndrome [45, 56]'.

The aetiology remains debated, but hypotheses include exposure to organophosphate nerve agents, pesticides, pyridostigmine bromide prophylaxis and other environmental toxins encountered during deployment.

### 3.9. Terrorist and Non-State Use of Chemical Weapons

Beyond state-sponsored warfare, chemical agents have been repeatedly employed by terrorist organisations, cults and individuals. Early examples include the Peoples Temple Cult mass suicide in Guyana, in which more than 900 people ingested cyanide-laced drinks [9, 20], and the 1982 Tylenol tampering in the United States, which led to seven deaths and prompted the adoption of tamper-proof pharmaceutical packaging [1]. Smaller scale attacks, such as pesticide poisonings in the Philippines (1987) and chlorine use by the Liberation Tigers of Tamil Eelam in Sri Lanka (1990), underscore the accessibility of readily available toxicants for mass harm [57, 58].

The most significant nonstate campaign was carried out by the Japanese cult Aum Shinrikyo between 1990 and 1995. The group conducted multiple assassination attempts and attacks using sarin, VX and phosgene oxime. The first large-scale release occurred in Matsumoto in 1994, where sarin exposure affected 600 people, killing seven [51, 59, 60]. This was followed by the infamous Tokyo subway attack in March 1995, in which coordinated sarin releases injured more than 6000 commuters, hospitalised nearly 1000 and killed 13. Detailed forensic reconstructions [17, 19] traced the synthesis and release of sarin, while investigative and government reports [22, 40, 51] confirmed Aum Shinrikyo's culpability. Subsequent epidemiological reports [61, 62] documented the long-term neuropsychiatric sequelae among survivors.

Sarin, like other G-series nerve agents, exerts its effects by inhibiting acetylcholinesterase, producing uncontrolled cholinergic stimulation that manifests as miosis, convulsions, respiratory failure and death [63]. Survivors often experience persistent neurological and psychiatric sequelae. These events marked the first major demonstration of a nonstate actor successfully synthesising and deploying military-grade nerve agents in an urban environment. The group then used phosgene oxime in a covert attack against a journalist, causing one injury [64]. The group later used VX in targeted attacks, causing multiple fatalities [64]. VX's lipophilicity and potency make it far more persistent than sarin, and even minute dermal exposures can cause lethal peripheral nervous system paralysis [65].

Other notable terrorist or criminal uses included the Fuerzas Armadas Revolucionarias de Colombia (FARC) tear gas grenade assault in Colombia (1999), which killed 65 people [66], and several religiously motivated poisonings in Africa and Asia, such as the tea poisonings in Uganda (2000) and Zimbabwe (2002) that caused mass fatalities [67]. In 2002, Chechen separatists used a fentanyl derivative during the Moscow theatre siege; while intended to be an incapacitant, the opioid's mechanism of action, the *μ*-opioid receptor agonism, led to central respiratory depression contributing to 125–205 deaths due to delayed disclosure of its identity to medical responders [1, 21, 68].

Taken together, these cases highlight several themes: the ease with which industrial and pharmaceutical chemicals can be repurposed as weapons; the ability of motivated groups to acquire and use sophisticated nerve agents; and the unique challenges of attributing and responding to non-state chemical attacks. They underscore that chemical warfare is not limited to state actors.

### 3.10. Chemical Terrorism in Iraq and Afghanistan (2003–2016)

In the post-2003 era, multiple terrorist groups used chemical agents in Iraq and Afghanistan, with varying levels of sophistication and effectiveness. Early incidents included improvised devices incorporating sulphur, sarin or mustard agents, though most failed to cause significant casualties [38]. The most consequential phase began in 2006–2007, when insurgents in Iraq conducted a series of 15 chlorine attacks and truck bombings across urban centres, killing 115 and injuring more than 850 people [38]. Al Qaeda also carried out three chlorine-based bombings in 2007, which killed 23 and injured 380 [57]. These attacks demonstrated how industrial chemicals could be repurposed for large-scale urban terror campaigns.

In Afghanistan, attacks increasingly targeted civilian and educational institutions. In 2009, an unknown gas attack on a girls' school injured more than 200 students [57]. Between 2010 and 2013, insurgents carried out dozens of coordinated poisonings, primarily using pesticides or suspected rodenticides. More than 2600 schoolchildren and staff were affected across these incidents, which underscored the vulnerability of soft civilian targets to readily available toxicants [38].

The Islamic State of Iraq and Syria (ISIS) further escalated chemical terrorism between 2014 and 2016, employing chlorine and sulphur mustard against both military and civilian populations. Documented incidents include chlorine attacks on Kurdish fighters, a 2016 strike in Kirkuk that injured 600 civilians and killed one, and large-scale plumes from burning sulphur stockpiles that injured 1500 people [38, 69]. These episodes highlighted ISIS's capacity to adapt conventional warfare with chemical agents and to integrate toxic materials into psychological and tactical operations.

The period between 2003 and 2016 illustrated how terrorist organisations increasingly exploited industrial chemicals and pesticides for asymmetric warfare. While crude compared to state-level programmes, these attacks caused thousands of injuries, instilled fear in civilian populations and reinforced the continuing risk of improved CWA dissemination in conflict zones.

### 3.11. Assassinations and Targeted Poisonings

Chemical agents have also been widely used in covert assassinations, often reflecting state involvement and the adaptability of toxicants to clandestine operations. During the Cold War, Soviet agents employed concealed delivery devices in Munich, killing Ukrainian dissidents Lev Rebet (1957) and Stepan Bandera (1959) with cyanide gas [9, 70]. The 1978 assassination of Bulgarian exile Georgi Markov in London with a ricin-tipped umbrella became an iconic case of chemical weapon use in espionage, while a similar ricin attack on Vladimir Kostov in Paris was survived. Bulgarian intelligence methods used in these assassinations have been described in detail [19, 22], while subsequent investigations and forensic analyses [70] detail the subcutaneous ricin pellet mechanism that led to Kostov's survival after surgical removal.

In the post-Soviet era, Russian agents and affiliates have continued to rely on chemical and toxicological methods. Cases include the fatal poisoning of banker Ivan Kivelidi with cadmium salts (1995), the dioxin poisoning of Ukrainian Viktor Yuschenko (2004) and the death of whistleblower Alexander Perepilichny from ingestion of *Gelsemium elegans.* Toxicological investigations [9, 21] first identified the alkaloid *gelsemine* as the causative agent, while forensic case reports [71–73] later confirmed its deliberate administration in several high-profile assassinations demonstrating renewed interest in plant-based toxins for covert operations.

The Novichok nerve agent, developed during the Cold War, re-emerged prominently in assassination attempts: against Bulgarian businessman Emilian Gebrev (2015), North Korean exile Kim Jong-nam with VX in Malaysia (2017), former double agent Sergei Skripal and his daughter in Salisbury, United Kingdom (2018), and Russian opposition leader Alexei Navalny [36, 42, 71]. These attacks demonstrated the persistence of military-grade nerve agents in modern intelligence operations and their capacity to cause both acute fatalities and long-term neurological sequelae.

More recent poisonings linked to Russian security services include those of dissident Vladimir Kara-Murza (2015 and 2017), journalist Elena Kostyuchenko (2022), politician Elvira Vikhareva (2022) and other opposition figures during the Russia–Ukraine conflict [74–76]. Reports also describe suspected poisonings of Georgian ex-president Mikheil Saakashvili and Ukrainian negotiators in 2022, underscoring the ongoing use of toxicants as tools of political suppression [77, 78].

Although not state-sponsored, serial poisonings, such as the cyanide murders by Jolly Joseph in India (2002–2016), illustrate that chemical agents can also be exploited for targeted killings in domestic contexts [79].

Assassination cases reveal several toxicological and operational patterns: reliance on highly potent agents (ricin, cyanide, Novichok and VX); novel delivery systems (umbrellas, disguised sprays and contaminated food and drink); and recurring attribution to state security devices. These events highlight the dual role of CWAs as both battlefield weapons and instruments of covert political violence.

### 3.12. The Syrian Civil War (2011–2023)

The Syrian Civil War has been the most intensively documented case of chemical warfare in the twenty-first century. Between March 2011 and November 2023, at least 222 chemical attacks were confirmed, with the vast majority (*n* = 217) attributed to the Syrian regime and five to ISIS. Analyses by the OPCW and United Nations investigators [1, 21] attribute the majority of Syrian attacks to regime forces, whereas reports by Human Rights Watch and Conflict Armament Research [27, 32, 66] document smaller scale deployments by ISIS; subsequent satellite and medical data analyses [80–82] substantiate these findings through independent verification of munitions and exposure symptoms.

The primary agents used were chlorine, sarin and mustard gas, reflecting both the accessibility of industrial chlorine and the continued stockpiling of legacy warfare agents.

These attacks caused at least 1,514 deaths and over 11,200 injuries [83], although the true numbers are likely higher, given underreporting in conflict zones. Chlorine, often deployed via barrel bombs or improvised munitions, produced pulmonary toxicity, while sarin exposures caused mass casualties through cholinergic crisis. The repeated use of these agents against civilian populations demonstrated both the persistence of state-level CWA deployment despite international prohibitions and the limitations of enforcement mechanisms under the CWC.

### 3.13. Recent Incidents (2022–2023)

In the past two years, chemical weapons and toxic agents have continued to feature in both conflict and asymmetric violence. Between December 2022 and April 2023, a series of 358 coordinated gas attacks on girls' schools in Iran sickened more than 3,000 students, raising concerns about the deliberate use of toxicants to instil fear and disrupt education [84]. Similar incidents occurred in Afghanistan, where two gas attacks on schools in Sar-e-Pul Province in June 2023 injured over 80 girls [85]. These events underscore the vulnerability of civilian targets and the psychological impact of chemical incidents, regardless of the scale of physical harm.

During the ongoing Russian full-scale invasion of Ukraine, reports have suggested the battlefield use of chemical agents, including chloropicrin, an early twentieth-century lachrymatory and pulmonary irritant [86, 87]. This highlights the persistence of legacy agents in modern conflicts, often repurposed for tactical disruption.

Elsewhere, allegations emerged during the October 2023 Israel–Hamas conflict that Israeli forces employed white phosphorus munitions, a substance with severe incendiary and toxic properties when used in populated areas [88].

These recent events demonstrate that chemical weapons remain a contemporary threat. They are not only used in large-scale conflict but also as tools of intimidation against civilians, perpetuating both acute and toxicological harm and long-term psychological trauma.

## 4. Conclusion

This study provides a systematic review of publicly known CWA utilisation in war and terrorist events from its first known use in 10,000 BCE to the present day. A limitation of our approach is that we did not systematically include journalistic or OSINT reports, which may capture otherwise undocumented or classified events. While such sources can provide valuable early signals, their reliability and verification present challenges. A further limitation of this review is the reliance on heterogeneous historical records, which vary in reliability and completeness. Although we applied a structured adjudication process and prioritised corroborated, peer-reviewed accounts, residual uncertainty in the attribution and accuracy of some events remains unavoidable.

Despite the ratification of the CWC in 1997, it is evident that not all actors in this area have either destroyed their stockpiles or have been dissuaded from their use. Chemical warfare remains a current and serious concern, at a local and global level, notably in light of recent and potential use during current armed conflicts. The question remains open as to how chemical weapons will play a role in future conflicts. Increasing surveillance of countries with a history of chemical weapon use may deter the development of more chemical weapons but is unlikely to stop nations covertly carrying out chemical warfare. Novel technologies are reducing barriers to widespread dissemination of chemical agents, such as drones.

From a toxicological perspective, the lessons of history emphasise the importance of preparedness for both acute and long-term health consequences. Agents, such as chlorine and phosgene, cause rapid pulmonary injury, whereas sulphur mustard and organophosphorus nerve agents are associated with chronic sequelae, including carcinogenesis, neurotoxicity and reproductive impairment [89]. The emergence of Novichok agents, with their high potency and persistence, highlights the continuing evolution of threats. Prevention of harms from CWAs relies on supporting and promoting global norms rejecting development, stockpiling and use of chemical weapons. Effective defence against CWAs therefore requires not only early detection and prompt identification of casualties, but also robust systems for triage, and the medical community must be prepared to diagnose and treat chemical warfare casualties rapidly, mass decontamination and sustained long-term medical monitoring of survivors. Finally, an integrated global surveillance system for chemical incidents would support these efforts.

## Figures and Tables

**Table 1:** History of chemical agent use in warfare.

Year/period	Chemical class	Chemical agent (common name)/weapon	Geospatial coordinates	Country	City	Event description and target	Responsible party (suspected)	Mode of delivery and source	Deaths	Injuries	References
10,000 BCE	Unknown	Unknown	30.5595°S, 22.9375°E	South Africa	Not stated	Unknown	South Africa	Wood, bone and stone arrowheads were dipped into poisons obtained from scorpion and snake venom, as well as poisonous plants	Unknown	Unknown	[1]

2000 BCE	Unknown	Unknown	20.5937°N, 78.9629°E	India	Not stated	Battle	India	Toxic sleep-inducing fumes that produced smoke screens	Unknown	Unknown	[14–16]

1000 BCE	Blistering agent	Arsenic sulphides	35.8617°N, 104.1954°E	China	Not stated	Unknown	China	Poisonous, noxious and irritant vapours called ‘soul-hunting fog'	Unknown	Unknown	[14, 17]

600 BCE	N/A	Rye ergot	34.5338°N, 43.4837°E	Mesopotamia	Not stated	Assyrians attacking enemies	Assyrians	Poisoned the water supply of enemies with fungi	Unknown	Unknown	[1]

590–600 BCE	Toxin	Protoanemonin	39.0742°N, 21.8243°E	Greece	Not stated	First Sacred War—Siege of Kirrha/Cirrha	Greece	Water contamination using Helleborus roots	Unknown	Unknown	[14, 18, 19]
	N/A	Steroidal saponins									
	N/A	Bufadienolides									

431–404 BCE	Blistering agent	Arsenic sulphides	39.0742°N, 21.8243°E	Greece	Not stated	Peloponnesian War—Siege of Plataea and Delium	Greece	Ignited pitch and sulphur generating arsenic smoke	Unknown	Unknown	[14, 17, 19–22]

Fourth century BCE	N/A	Unknown	35.8617°N, 104.1954°E	China	Not stated	The Mohist sect in China used bellows to pump smoke from burning balls into tunnels being dug by a besieging army	China	Smoke pump derived from plants and toxic vegetables producing a mustard	Unknown	Unknown	[1]

200 BCE	N/A	Unknown	26.0198°N, 32.2778°E	Tunisia (Ancient Carthage)	Not stated	The Carthaginians spiked wine to sedate enemies, then returned once the enemy was sleeping to kill them	Carthaginians	Wine contamination using mandrake root	Unknown	Unknown	[14, 19]

200 BCE	Unknown	Unknown	39.0742°N, 21.8243°E	Greece	Ambracia	War	Greece	Toxic smoke	Unknown	Unknown	[14]

82–72 BCE	Unknown	Unknown	39.0742°N, 21.8243°E	Greece	Not stated	War	Greece	Toxic smoke	Unknown	Unknown	[14, 20]

50 CE	Blood agent	Hydrogen cyanide (AC)	41.8719°N, 12.5674°E	Italy	Not stated	Defence of the Roman Empire	Italy	Contaminated water using cherry laurel	Unknown	Unknown	[1]

46–120 CE	Unknown	Unknown	39.0742°N, 21.8243°E	Greece	Not stated	Unknown	Greece	Toxic smoke	Unknown	Unknown	[1]

1000	Unknown	Aconitum	45.4507°N, 68.8319°E	Mongolia	Not stated	Unknown	Mongols	Gas bombs made from sulphur, nitre (saltpetre), oil, aconite, powdered charcoal, wax and resin	Unknown	Unknown	[1]

660	Unknown	Greek fire	39.0742°N, 21.8243°E	Greece	Not stated	Callinicus of Heliopolis protecting the Greek Empire using ‘Greek fire'	Greece	Pumped through a siphon or large syringe and used by hand from handmade grenades. Thought to be made from naphtha, nitre (saltpetre), sulphur, petroleum and quicklime sulphur; or a mixture of phosphorus and saltpetre	Unknown	Unknown	[1]

674–677	Unknown	Greek fire	39.0742°N, 21.8243°E	Greece	Not stated	The Arab siege of Constantinople using ‘Greek fire'	Greece	As above	Unknown	Unknown	[1, 20]

717–718	Unknown	Greek fire	39.0742°N, 21.8243°E	Greece	Not stated	The Battle of Syllaeum using ‘Greek fire'	Greece	As above	Unknown	Unknown	[1]

941	Unknown	Greek fire	39.0742°N, 21.8243°E	Greece	Not stated	Attacks against Muslims using ‘Greek fire'	Greece	As above	Unknown	Unknown	[1]

941	Unknown	Greek fire	41°00′55″N 28°59′05″E	Turkey	Byzantium/Constantinople	The Byzantines used ‘Greek fire' against the Vikings and against the Venetians during the Fourth Crusade	Byzantines	As above	Unknown	Unknown	[1]

1043	Unknown	Greek fire	39.0742°N, 21.8243°E	Greece	Not stated	Defending against Russian attacks	Greece	As above	Unknown	Unknown	[1]

960–1279 AD	Blistering agent	Arsenic sulphides	35.8617°N, 104.1954°E	China	Not stated	Battle	China	Toxic smoke derived from a metalloid in the natural environment	Unknown	Unknown	[19, 90, 91]

1155	Unknown	Unknown	51.1657°N, 10.4515°E	Germany	Not stated	Unknown	Unknown	Toxic smoke	Unknown	Unknown	[1]

1456	Blistering agent	Arsenic sulphides	44.0165°N, 21.0059°E	Serbia	Belgrade	Defence of the country from the Turkish	Serbia	Toxic smoke	Unknown	Unknown	[23]

Fifteenth century	Unknown	Unknown	45.4408°N, 12.3155°E	Italy	Venice	Attacks on wells, crops and animals	Unknown	Poison-filled mortar shells and poison chests	Unknown	Unknown	[1]

Sixteenth century	Unknown	Unknown	45.4408°N, 12.3155°E	Italy	Venice	Attacks on wells, crops and animals	Unknown	Poison-filled mortar shells and poison chests	Unknown	Unknown	[1]

1618–1648	Unknown	Unknown	51.1657°N, 10.4515°E	Germany	Not stated	Thirty Years' War	Germany	Toxic smoke projectiles derived from shredded hoofs, horns and asafoetida mixed with pitch	Unknown	Unknown	[14, 26, 91]

1672	N/A	Belladonna alkaloids	51.1657°N, 10.4515°E	Netherlands	Groningen	Siege of Groningen	Netherlands	Toxic fumes from explosive and incendiary devices derived from the plant Atropa belladonna	Unknown	Unknown	[1, 14, 19]

1854–1856	N/A	Sulphur	48.3794°N, 31.1656°E	Ukraine	Sevastopol	Attack against the Russians	United Kingdom	Toxic fumes	Unknown	Unknown	[24]

1899	N/A	Picric acid	30.5595°S, 22.9375°E	South Africa	Not stated	Used by British soldiers during the Boer War	United Kingdom	Shells	Unknown	Unknown	[1, 14, 19, 22–24]

1904–1905	Blistering agent	Arsenic sulphides	49°36′ 0″ N, 117°25′ 58″ E	China	Manchuria	The Russo-Japanese War	Imperial Japan	Rag torches on bamboo poles	Unknown	Unknown	[1, 40]

August 1914	Tear gas	Ethyl bromoacetate	50°49′10.19″ N 2°55′32.39″ E	France	Not stated	Used by French forces—World War I	France	Grenades filled with tear gas	See below	See below	[19, 26, 27]

October 1914	Tear gas	Niespulver	50°49′10.19″ N 2°55′32.39″ E	France	Neuve Chapelle	Used by German forces against the British—World War I	Germany	Propellant charge	See below	See below	[19, 26]
	N/A	Dianisidine									
	N/A	Chlorosulfate									

November 1914	Tear gas	Chloroacetone	50°49′10.19″ N 2°55′32.39″ E	France	Not stated	Used first by French forces—World War I	France	Gas cylinder	See below	See below	[19, 26]

January 1915	Tear gas	Xylyl bromide	51.9194°N, 19.1451°E	Poland	Borzymow	Used by German forces during the Battle of Humin-Bolimov against the Russians—World War I	Germany	Gas cylinder	See below	See below	[1, 19, 20, 26, 36]

April 1915	Pulmonary agent	Chlorine gas	50.5039°N, 4.4699°E	Belgium	Ypres	Used by the German force against the British, French and Canadian soldiers during the Battle of Ypres in Flanders—World War I	Germany	Gas cylinder	See below	See below	[20, 21, 23, 24, 36]

April and December 1915	Pulmonary agent	Phosgene oxime	50.5039°N, 4.4699°E	Belgium	Ypres	Used by the German force against the British Troops during the Battle of Ypres—World War I	Germany	Gas cylinder	See below	See below	[19, 21, 22, 24, 26, 27, 33, 34]
	Pulmonary agent	Diphosgene									

July 1916	Blood agent	Hydrogen cyanide (AC)	46.2276°N, 2.2137°E	France	Not stated	Used by French forces—World War I	France	Gas cylinder	See below	See below	[19, 26]

October 1916	Blood agent	Cyanogen chloride	46.2276°N, 2.2137°E	France	Not stated	Used by French forces—World War I	France	Gas cylinder	See below	See below	[17, 19, 26, 32–34, 92]

July 1917	Vomiting agent	Diphenylchlorarsine (Clark 1)	51.1657°N, 10.4515°E	German Empire	Not stated	World War I	German Empire	Gas cylinder	See below	See below	[1, 19, 26]

July 1917	Blistering agent	Diphenylchlorarsine (Clark 1)	51.1657°N, 10.4515°E	German Empire	Not stated	World War I	German Empire	Gas cylinder	See below	See below	[19, 26]

July 1917	Blistering agent	Sulphur mustard	51.1657°N, 10.4515°E	German Empire	Not stated	Against the French, British and Russians—World War I	German Empire	Gas cylinder	See below	See below	[1, 20, 21, 24, 33, 34]

March 1918	Blistering agent	Methyldichloroarsine	51.1657°N, 10.4515°E	German Empire	Not stated	World War I	German Empire	Gas cylinder	See below	See below	[19, 26]

1914–1918	N/A	Acrolein	51.1657°N, 10.4515°E	German Empire	Not stated	World War I	German Empire	Gas cylinder	90,000	1,300,000	[1, 4, 9, 34]
	Tear gas	Bromoacetone									
	Tear gas	Bromobenzyl cyanide									

1919	Blistering agent	Sulphur mustard	61.5240°N, 105.3188°E	Soviet Union	Not stated	The British Army attacking the Red Russian Army during the Russian Civil War	United Kingdom	Unknown	Unknown	Unknown	[9, 40]
	Tear gas	Adamsite (DM)									
	Blistering agent	Diphenylchlorarsine (Clark 1)									

May 1921	Unknown	Unknown	52.7371°N, 41.4396°E	Soviet Union	Tambov	The Red Army attacking peasant rebels and civilians during the Russian Civil War	Soviet Union	Unknown	Unknown	Unknown	[9]

1921–1926	Blistering agent	Sulphur mustard	31.7917°N, 7.0926°W	Morocco	Rif region	Attacks on Riffians during the Rif War	Spain	Shells and bombs	Unknown	Unknown	[9, 40]
	Tear gas	Bromomethyl ethyl ketone									
	Pulmonary agent	Chloropicrin									

1934–1937	Blistering agent	Sulphur mustard	43.7934°N, 87.6271°E	China	Xinjiang	Attacks on Chinese soldiers	Russian Federation	Unknown	Unknown	Unknown	[9]

1935–1936	Tear gas	Chloroacetophenone (CN)	9.1450°N, 40.4897°E	Ethiopia (Abyssinia)	Not stated	Second Italo-Ethiopian War	National Fascist Party of Italy led by Mussolini	Grenades and bombs	15,000	Unknown	[1, 19–21, 23, 24, 26, 40]
	Blistering agent	Sulphur mustard									
	Pulmonary agent	Chlorine gas									
	Blistering agent	Diphenylchlorarsine (Clark 1)									
	Pulmonary agent	Phosgene oxime									

1937–1945	Tear gas	Chloroacetophenone (CN)	35.8617°N, 104.1954°E	China (Manchuria)	Not stated	Invasion by Imperial Japan—Second Sino-Japanese War	Empirical Japan	Aircraft bombs, artillery shell and toxic candles	10,000	72,000	[19, 20, 23, 24, 26, 27, 40, 93]
	Vomiting agent	Diphenylcyanoarsine (Clark II)									
	Nettle agent	Carbon tetrachloride									
	Blistering agent	Sulphur mustard									
	Blistering agent	Lewisite									
	Blood agent	Hydrogen cyanide (AC)									
	Blistering agent	Diphenylchlorarsine (Clark 1)									
	Pulmonary agent	Phosgene oxime									

8 September 1939	Blistering agent	Sulphur mustard	49.7446°N, 21.4723°E	Poland	Jaslo	Polish resistance attacking German troops	Poland	Unknown	2	12	[9]

December 1941 to April 1945	Blood agent	Hydrogen cyanide (AC)	51.1657°N, 10.4515°E	Nazi Germany	Oranienburg	Concentration camps (Sachsenhausen, Mauthausen and Auschwitz)—World War II	Germany, Poland and Austria	Pellets used in gas chambers	1,600,000	Unknown	[1, 17, 19–22, 24, 26, 27, 29, 33]
	Pulmonary agent	Carbon monoxide	51.9194°N, 19.1451°E	Poland	Oswiecim						
			47.5162°N, 14.5501°E	Austria	Mauthausen						

April 1946	Blistering agent	Arsenic sulphides	50.1186°N, 9.8918°E	Germany	Hammelburg	Jewish Holocaust survivors poisoned German prisoners of war in Stalag 13	Nakam (meaning ‘revenge' in Hebrew)	Unknown	Unknown	2283	[9]

12 October 1957	Blood agent	Hydrogen cyanide (AC)	48.1351°N, 11.5820°E	Germany	Munich	Assassination of Lev Rebet	Russian agents	Poison atomiser mist gun	1	None	[19, 26, 70]

15 October 1959	Blood agent	Hydrogen cyanide (AC)	48.1351°N, 11.5820°E	Germany	Munich	Assassination of Stepan Bandera	Russian agents	Chemical dust spraying gun	1	None	[14, 70]

1962–1975	Tear gas	Used by the United States and South Vietnam: 2-chlorobenzalmalononitrile (CS)	14.0583°N, 108.2772°E	Vietnam	Not stated	Vietnam War	The United States, South Vietnam, Vietnam and Laos	Aerial dissemination	An estimated 1,353,000 deaths were recorded during the war period; however, true deaths due to chemical warfare remain unknown	Unknown	[9, 19–21, 26, 40, 45]
			19.8563°N, 102.4955°E	Laos							
	Tear gas	Used by South Vietnam: Adamsite (DM)	12.5657°N, 104.9910°E	Cambodia							
	Herbicide	Used by the United States: Agent Purple, Orange, White and Blue containing various mixtures of *n*-butyl 2,4-dichlorophenoxyacetate (2,4-D), *n*-butyl 2,4,5-trichlorophenoxyacetate (2,4,5-T), *iso*-butyl trichlorophenoxyacetate, cacodylic acid or picloram									

1963–1967	Unknown	Unknown irritants	15.5527°N, 48.5164°E	Yemen	Not stated	Intervention by Egypt against Yemeni Royalists—Yemen Civil War	Egypt	Aircraft bombs	1400	900	[9, 19, 20, 24, 26, 40, 47]
	Blistering agent	Sulphur mustard									
	Tear gas	Unknown									
	Nerve agent	Unknown									

1976–1978	Pesticide	Parathion	19.0154°S, 29.1549°E	Rhodesia (Zimbabwe)	Not stated	During the Rhodesian Civil War, the embattled regime of Ian Smith used multiple chemical and biological agents against the guerrillas	Ian Smith	Poisoning wells and infecting clothing of terrorists	Unknown	Unknown	[9, 48]
	N/A	Thallium									

7 September 1978	Toxin	Ricin	42.7339°N, 25.4858°E	United Kingdom	London	Assassination of Bulgarian Georgi Markov	Unknown	Umbrella	1	None	[19, 21, 70, 94–96]

18 November 1978	Blood agent	Hydrogen cyanide (AC)	4.8604°N, 58.9302°W	Guyana	Jonestown	Mass suicide by cult members of the Peoples Temple Cult	Peoples Temple Cult	Injection	913	Unknown	[9, 20]

1975–1983	Toxin	Unknown	19.8563°N, 102.4955°E	Laos	Not stated	Lethal and incapacitating chemicals and toxins against Hmong resistance and soldiers over 261 instances	Vietnamese and Laos troops	Aerial burst rockets and spraying	6504	Unknown	[9, 49]
	Unknown	Unknown									

1978–1983	Unknown	Unknown	12.5657°N, 104.9910°E	Cambodia	Western Kampuchea	124 attacks against rebel forces	Vietnamese military	Unknown	1014	Unknown	[9]

1979–1989	Nerve agent	Unknown	33.9391°N, 67.7100°E	Afghanistan	Not stated	Invasion of Afghanistan	Russia	Unknown	Unknown	Unknown	[9]
	Pulmonary agent	Phosgene oxime									
	Tear gas	Unknown									

1982	Blood agent	Hydrogen cyanide (AC)	37.0902°N, 95.7129°W	United States	Chicago	Product tampered with and then returned to the offending store and sold to the public	Unknown	Capsules laced with chemicals	7	Unknown	[1]

1980–1988	Blistering agent	Sulphur mustard	32.4279°N, 53.6880°E	Iran	Not stated	Iran–Iraq War	Iran and Iraq	Aircraft bombs	An estimated 700,000 lives were lost with 30,000 Iranians due to chemical weapons	Unknown	[17–21, 27, 29, 32, 40, 50–52, 69, 97]
	Nerve agent	Tabun									
	Nerve agent	Sarin									
	Tear gas	2-chlorobenzalmalononitrile (CS)	33.2232°N, 43.6793°E	Iraq							

6 September 1987	Pesticide	Unknown pesticides	12.8797°N, 121.7740°E	Philippines	Zamboanga City	Unknown terrorist group attacking police	Unknown	Water supply contamination	19	140	[57]

January 1987	Blistering agent	Sulphur mustard	15.4542°N, 18.7322°E	Chad	Not stated	Attacks on Chad	Libya	Unknown	Unknown	Unknown	[1]

16 March 1988	Blistering agent	Sulphur mustard	33.2232°N, 43.6793°E	Iraq (Kurdistan)	Halabja	Campaign against Kurdistan—Halabja Massacre	Iraq	Aircraft bombs	5000	10,000	[19, 21, 27, 53–55]
	Nerve agent	Sarin									

15 June 1990	Pulmonary agent	Chlorine gas	7.8731°N, 80.7718°E	Sri Lanka	East Kiran	Attack on a Sri Lankan military base	Liberation Tigers of Tamil Islam	Unknown	1	40	[57]

1990–1991	Nerve agent	Tabun	29.3117°N, 47.4818°E	Kuwait		Invasion of Kuwait by Iraq during the Persian Gulf War led to the deployment of U.S. military personnel and Coalition countries entering	Iraq	Destroying of chemical weapon stockpiles	None	700,000	[45, 98]
	Nerve agent	Sarin									

26 February 1993	Blood agent	Hydrogen cyanide (AC)	37.0902°N, 95.7129°W	United States	New York	Attempted bombing on the World Trade Centre	Ramzi Yousef, Mahmud Abouhalima, Mohammad A. Salameh, Nidal Ayyad, Abdul Rahman Yasin and Ahmed Ajaj	Bomb	6		[1, 20]

1 January 1994	Blood agent	Hydrogen cyanide (AC)	38.8610°N, 71.2761°E	Tajikistan	Dushanbe	Attack against Russian forces	Tajik opposition	Poisoned champagne by Tajik opposition	5	53	[57]

21 January 1994	Unknown	Unknown	36.6322°N, 33.6176°E	Turkey	Ormancik	Terrorist attack against civilians	Unknown	Chemical grenades	16	Unknown	[19, 61]

27 June 1994	Nerve agent	Sarin	36.2380°N, 137.9720°E	Japan	Matsumoto	Terrorist attacks to kill judges who were expected to rule against the cult in a lawsuit concerning a real estate dispute	Aum Shinrikyo	Gas-filled plastic bags	9	600	[17, 19–22, 29, 40, 51, 61, 66]

20 March 1995	Nerve agent	Sarin	35.6764°N, 139.6500°E	Japan	Tokyo	Attacks on commuters from five trains travelling towards Kasumigaseki station	Aum Shinrikyo	Gas-filled plastic bags	12	5000	[17, 19–22, 29, 40, 51, 61, 62, 66]

5 May and 4 July 1995	Blood agent	Hydrogen cyanide (AC)	35.6764°N, 139.6500°E	Japan	Tokyo	Attacks on commuters on a Tokyo subway, though this attempt failed	Aum Shinrikyo	Unknown	None	None	[9]

4 August 1995	Nerve agent	Novichok	61.5240°N, 105.3188°E	Russian Federation	Not stated	Assassination of Russian banker Ivan Kivelidi and his secretary Zara Ismailova	Russian agents	Poisoning of tea	2	None	[70]

December 1994 and January 1995	Nerve agent	VX	35.6764°N, 139.6500°E	Japan	Tokyo	Attempted to assassinate targets	Aum Shinrikyo	Unknown	1	2	[9, 66]

12 December 1999	Tear gas	Unknown	7.1026°N, 77.7620°W	Colombia	Jurado	Attack on naval base and army barracks	FARC	Grenades	65	Unknown	[66]

17 March 2000	Unknown	Unknown	0.1699°N, 30.0781°E	Uganda	Kasese	Attack on supporters of the ‘Movement for the Restoration of God's Ten Commandments'	Unknown	Poisoning of tea	Unknown	Unknown	[57]

21 April 2002	Unknown	Unknown poison	48.7080°N, 44.5133°E	Russian Federation	Volgograd	Assassination of Lecha Ismailov, a Chechen guerrilla commander	Russian agents	Poisoned tea	1	None	[71]

26 October 2002	N/A	Fentanyl derivative	61.5240°N, 105.3188°E	Russian Federation	Moscow	Russians used incapacitant to disable Chechen terrorists who took control of a large theatre	Russian agents	Gassing of room	244	Unknown	[1, 21]

23 May 2002	Pesticide	Unknown pesticides	19.0154°S, 29.1549°E	Zimbabwe	Manicaland Province	Attack on supporters of the ‘Johanna Marange Apostolic Church'	Unknown	Poisoning of tea	7	47	[57]

11 November 2002	Unknown	Unknown poison	29.0316°N, 111.6985°E	China	Changde	Unknown criminal organisation poisoned a high school	Unknown	Poisoning of food	Unknown	193	[9]

2002–2016	Blood agent	Hydrogen cyanide (AC)	10.1632°N, 76.6413°E	India	Koodathayi	Jolly Joseph poisoned her family members to cover her lies and gain power	Jolly Joseph	Poisoning of food and liquids	6	None	[79]

June–July 2003	Blistering agent	Sulphur mustard	36.3489°N, 43.1577°E	Iraq	Mosul	Terrorist attack against civilians and U.S. soldiers	ISIS	Sulphur stockpiles at mine set on fire	None	41	[38]

15 May 2004	Nerve agent	Sarin	33.3152°N, 44.3661°E	Iraq	Baghdad	Terrorist attack against U.S. soldiers	ISIS	Improvised explosive device using chemical weapon artillery shell near airport (failed launch)	None	2	[38]

5 September 2004	N/A	Dioxin	48.3794°N, 31.1656°E	Ukraine	Not stated	Assassination attempt on Viktor Yushchenko at a dinner with a group of senior Ukrainian officials	Russian agents	Poisoned food	None	1	[9, 21, 71]

11 September 2004	Unknown	Unknown poison	61.5240°N, 105.3188°E	Russian Federation	Not stated	Assassination of Roman Tsepov	Russian agents	Poisoned tea	1	None	[9, 72]

25 September 2006	Blistering agent	Sulphur mustard	33.3152°N, 44.3661°E	Iraq	Baghdad	Attack against U.S. soldiers	ISIS	Improvised explosive device using chemical weapon artillery shell	None	2	[38]

6 October 2006	Unknown	Unknown poison	33.3152°N, 44.3661°E	Iraq	Numaniyah	Terrorist attack against police	ISIS	Poisoning of food on police base	7	700	[38]

October 2006–June 2007	Pulmonary agent	Chlorine gas	32.5445°N, 45.4075°E	Iraq	Ramade, Bagdad, Falluja and others	Across 15 attacks against civilian targets	ISIS	Car/truck bombings with chlorine tanks	115	854	[38]

11 March 2007	Blistering agent	Sulphur mustard	33.2232°N, 43.6793°E	Iraq	Not stated	Terrorist attack against U.S. soldiers	ISIS	Artillery shells (failed attempt)	None	2	[38]

20 February 2007	Pulmonary agent	Chlorine gas	33.2232°N, 43.6793°E	Iraq	Taji	Terrorist attack against police and civilians	Al Qaeda	Suicide bombing trucks	4	140	[57]

12 March 2007	Pulmonary agent	Chlorine gas	33.2232°N, 43.6793°E	Iraq	Taji	Terrorist attack against police and civilians	Al Qaeda	Suicide bombing trucks	2	30	[57]

16 March 2007	Pulmonary agent	Chlorine gas	33.2232°N, 43.6793°E	Iraq	Taji	Terrorist attack against police and civilians	Al Qaeda	Suicide bombing trucks	350	None	[57]

2 April 2007	Pulmonary agent	Chlorine gas	33.2232°N, 43.6793°E	Iraq	Taji	Terrorist attack against police and civilians	Al Qaeda	Suicide bombing trucks	30	50	[57]

2009	Unknown	Unknown gas	33.9391°N, 67.7100°E	Afghanistan	Taji	Attacks on educational institutions	Taliban	Unknown	1	207	[57]

April–August 2010	Pesticide	Unknown pesticides	33.9391°N, 67.7100°E	Afghanistan	Kabul, Kunduz and others	Across 20 attacks against schoolchildren	ISIS	Gas cylinders	None	672	[38]

10 November 2012	N/A	*Gelsemium elegans*	51.3148°N, 0.5600°W	United Kingdom	Surrey	Assassination of Alexander Perepilichny, a former Russian banker whistleblower	Russian agents	Unknown	1	None	[9, 73]

March 2012–April 2013	Unknown	Rat poison	33.9391°N, 67.7100°E	Afghanistan	Not stated	Across nine attacks against civilian targets and police	ISIS	Poisoning of food	53	40	[9]

April 2012–June 2013	Pesticide	Unknown pesticides	33.9391°N, 67.7100°E	Afghanistan	Takhar Province, Sar-e-Pul Province and others	Across 23 attacks against schoolchildren	ISIS	Gas cylinders and water poisoning	Unknown	1952	[38]

March 2011 to March 2023	Pulmonary agent	Chlorine gas	33.5138°N, 36.2765°E	Syria	Damascus, Khan Al Asal, Sarib, Ghouta and Joba	Attacks against rebel soldiers and civilians—Syrian Civil War	Assad regime (Syrian Military)	Aerial bombing and rockets filled with chemical agents	500,000	Unknown	[1, 21, 27, 32, 33, 80, 82, 83, 99]
	Nerve agent	Sarin									
	Unknown	Unknown									
	Blistering agent	Sulphur mustard									

September –October 2014	Pulmonary agent	Chlorine gas	33.2232°N, 43.6793°E	Iraq	Duluiya and Balad	Islamic State terrorist attack against Iraqi and Shiite soldiers	ISIS	Aerial bombing	None	40	[100]
	Blistering agent	Sulphur mustard									

23 January 2015	Pulmonary agent	Chlorine gas	36.3489°N, 43.1577°E	Iraq	Mosul and Syrian border	Islamic State terrorist attack against Kurdish soldiers	ISIS	Truck bombing	Unknown	30	[69, 100]

26 May 2015	Unknown	Unknown poison	56.4884°N, 84.9480°E	Russian Federation	Moscow	Poisoning of Vladimir Kara-Murza, a Russian dissident with suspected dioxin	Russian security service agents	Potentially in food consumed at a restaurant	None	1	[74]

2015	Nerve agent	Novichok	42.7339°N, 25.4858°E	Bulgaria	Sofia	Attempted poisoning of Emilian Gebrev	Russian agents	Smeared on car door handles	None	1	[71]

8 March 2016	Blistering agent	Unknown	36.3489°N, 43.1577°E	Iraq	Taza and Kirkuk	Islamic State terrorist attack against civilians	ISIS	Unknown	1	600	[100]

21–27 October 2016	Blistering agent	Sulphur mustard	36.3489°N, 43.1577°E	Iraq	Mosul	Islamic State terrorist attack against civilians and soldiers	ISIS	Sulphur mine set on fire, producing widespread plumes	2	1500	[100]

2 February 2017	Unknown	Unknown poison	56.4884°N, 84.9480°E	Russian Federation	Moscow	Second poisoning of Vladimir Kara-Murza, a Russian dissident with suspected dioxin	Russian security service agents	Unknown	None	1	[74]

13 February 2017	Nerve agent	VX	4.2105°N, 101.9758°E	Malaysia	Kuala Lumpur	Poisoning of Kim Jongnam at Kuala Lumpur International Airport by Siti Aisyah an Indonesian woman and Doan Thi Huong, a Vietnamese woman	North Korea	Oily substance smeared on face	1	Unknown	[36, 42]

4 March 2018	Nerve agent	Novichok	51.0688°N, 1.7945°W	United Kingdom	Salisbury	Poisoning of Sergej, a former Russian military officer and double agent, and his daughter Yulia Skripal and Nicholas Baily, the first investigator to arrive	Russian agents	Perfume bottle	None	3	[71]

30 June 2018	Nerve agent	Novichok	51.1679°N, 1.7630°W	United Kingdom	Amesbury	Accidental poisoning of Charlie Rowley and Dawn Sturgess	Russian agents	Perfume bottle laced with agent believed to be used in Sergej Skripal poisoning	1	1	[71]

20 August 2020	Nerve agent	Novichok	56.4884°N, 84.9480°E	Russian Federation	Tomsk	Assassination attempt by poisoning of Alexei Navalny suspected to be by Russia	Russian agents	Chemical applied to underwear	None	1	[71]

October, 2021–2022	Unknown	Unknown poison	42.3154°N, 43.3569°E	Georgia	Tbilisi	Poisoning of Georgia's jailed ex-president, Mikhail Saakashvili by suspected heavy metals	Unknown	Suspected through medication administered after his hunger strike	None	1	[78]

March 2022	Unknown	Unknown poison	48.3794°N, 31.1656°E	Ukraine	Kyiv	Poisoning of Russian billionaire Roman Abramovich, Ukrainian lawmaker Rustem Umerov and another negotiator who were negotiating in Ukraine after the Russia–Ukraine invasion commenced	(suspected Russian security service agents)	Unknown	None	3	[77]

2 May 2022	Unknown	Unknown poison	49.8175°N, 15.4730°E	Czech Republic	Prague	Poisoning of President and founder of the Free Russia Foundation Natalia Arno	(suspected Russian security service agents)	Unknown	None	1	[101]

18 October 2022	Unknown	Unknown poison	51.1657°N, 10.4515°E	Germany	Munich	Poisoning of Russian journalist Elena Kostyuchenko from Novaya Gazeta and independent Russian newspaper	(suspected Russian security service agents)	Unknown	None	1	[75]

November 2022	N/A	Potassium dichromate	56.4884°N, 84.9480°E	Russian Federation	Moscow	Poisoning of Russian opposition politician Elvira Vikhareva	(suspected Russian security service agents)	Unknown	None	1	[76]

December 2022 to April 2023	Unknown	Unknown	32.4279°N, 53.6880°E	Iran	Not stated	Across 358 coordinated chemical attacks on girls' schools	Unknown	Unknown	Unknown	13,000	[84]

2022–2024	Herbicide	2-chlorobenzalmalononitrile (CS)	47.0971°N, 37.5434°E	Ukraine	Mariupol	Confirmed chemical attack with artillery barrages	Russian Federation	Unknown	Unknown	Unknown	[86, 87, 102]

June 2023	Unknown	Unknown poison	33.9391°N, 67.7100°E	Afghanistan	Sar-e-Pul Province	Two separate attacks on girls' schools	Unknown	Gas poisoning	None	80	[85]

October 2023	Unknown	White phosphorus	31.5017°N, 34.4668°E	Gaza Strip	Gaza	Retaliation attack on Hamas	Israel	Missiles	Unknown	Unknown	[88]

**Table 2:** Toxicological properties of selected major chemical warfare agents (alphabetical order).

Agent	Class	Origin	Mechanism of action	LD_50_/LC_50_	Environmental persistence	Acute effects	Chronic effects	References
2-Chlorobenzalmalononitrile (CS)	Tear gas	Synthetic	Potent sensory irritant; activates transient receptor potential ankyrin ion channels	LCt_50_ (inhalation) ∼50, mg min/m^3^ (rats)	Hours (aerosolised)	Lacrimation, cough and chest tightness	Chronic bronchitis and corneal scarring	[103, 104]

Adamsite (DM)	Tear gas	Synthetic (arsenical)	Irritant; triggers sensory nerves and respiratory mucosa	LCt_50_ ∼11,000 mg min/m^3^ (rats)	Days (stable solid)	Nausea, vomiting and chest pain	Chronic respiratory inflammation and neuropathy	[18, 105]

Agent Orange (2,3,7,8-tetrachlorodibenzo-p-dioxin: TCDD)	Herbicide	Synthetic (dioxins and phenoxyacetic acids)	Dioxin disrupts endocrine/gene regulation	LD_50_ (rat, oral) ∼50 µg/kg (TCDD)	Months–years	Dermatitis and gastrointestinal upset	Cancer, reproductive harm and endocrine disruption	[106, 107]

Arsenic sulphides	Blistering agent	Mineral	Protein binding and enzyme inhibition	LD_50_ ∼185 (orally) and 936 (dermally) mg/kg (rat)	Persistent	Burns and gastrointestinal upset	Skin/lung cancer and neuropathy	[108]

Chloroacetophenone (CN)	Tear gas	Synthetic	Transient receptor potential ankyrin activation	LCt_50_ ∼417–850 mg min/m^3^	Hours–days	Eye tearing and cough	Corneal damage and chronic bronchitis	[109]

Chlorine	Pulmonary agent	Industrial chemical	Hydrolyses to hypochlorous acid and hydrochloric acid, leading to oxidative injury	LC_50_ ∼293 ppm (30 min, rat)	Minutes–hours	Pulmonary oedema and hypoxia	Chronic bronchitis, reactive airways dysfunction syndrome and reduced lung function	[110]

Chloropicrin	Pulmonary agent	Synthetic pesticide	Deoxyribonucleic acid/protein alkylation	LCt_50_ ∼1500 mg min/m^3^	Days	Eye pain, pulmonary oedema and vomiting	Reactive airway disease and chronic lung fibrosis	[111, 112]

Cyanogen chloride (CK)	Blood agent	Synthetic	Cytochrome oxidase inhibition	LCt_50_ ∼11,000 mg min/m^3^	Minutes	Seizures, coma and death	Neurocognitive deficits and Parkinsonism-like syndromes	[113–115]

Diphenylchlorarsine (Clark 1)	Blistering agent	Synthetic (arsenical)	Irritant and arsenical toxicity	LCt_50_ ∼11,000 mg min/m^3^	Days	Vomiting, cough and chest pain	Neuropathy and chronic lung injury	[105, 116]

Diphenylcyanoarsine (Clark II)	Blistering agent	Synthetic (arsenical)	Irritant, arsenical toxicity and mucous membrane inflammation	LCt_50_ ∼11,000 mg min/m^3^ (human estimate)	Days	Sneezing, vomiting, chest pain and severe mucous irritation	Chronic bronchitis, neuropathy and possible arsenic-related carcinogenesis	[113]

Diphosgene	Pulmonary agent	Synthetic	Causes delayed pulmonary oedema via acyl chloride hydrolysis	LCt_50_ ∼3000 mg min/m^3^	Moderate persistence	Cough, choking and delayed-onset respiratory distress	Chronic bronchitis and pulmonary fibrosis	[31, 113]

Fentanyl derivative	Incapacitating opioid	Synthetic pharmaceutical	μ-opioid receptor agonist	LD_50_ ∼3 mg/kg (rat)	Hours	Coma, apnoea and death	Dependence, tolerance and cognitive impairment	[117]

Hydrogen cyanide (AC)	Blood agent	Synthetic/industrial	Cytochrome oxidase inhibition leading to histotoxic hypoxia	LCt_50_ ∼2500–5000 mg min/m^3^	Minutes	Seizures and cardiac arrest	Cognitive impairment and Parkinsonism-like sequelae	[114, 118]

Lewisite	Blistering agent	Synthetic (arsenical)	Binds to thiols to cause toxicity	LCt_50_ ∼200 mg min/m^3^ (human estimate)	Days–weeks	Blistering and pulmonary burns	Cancer risk, neuropathy and chronic skin lesions	[105, 118]

Novichok	Nerve agent	Synthetic (V-series, organophosphate)	Irreversible acetylcholinesterase inhibitor	LD_50_ ∼0.22 mcg/kg (human estimate)	Days–months	Cholinergic crisis	Long-term neurocognitive impairment and seizures	[119]

Phosgene	Pulmonary agent	Industrial precursor	Protein/lipid acylation	LCt_50_ ∼3200 mg min/m^3^ (human) LCt_50_ ∼32 ppm (30 min, rat)	Hours	Latent pulmonary oedema	Chronic lung fibrosis and emphysema	[113, 120]

Ricin	Protein toxin	Plant (castor beans; Ricinus communis)	Ribosome inactivation inhibiting protein synthesis	LD_50_ ∼3–5 µg/kg (inhalation) LD_50_ ∼20 mg/kg	Days	Gastrointestinal failure and multiorgan collapse	Organ fibrosis and chronic weakness	[121]

Sarin (GB)	Nerve agent	Synthetic (G-series)	Irreversible acetylcholinesterase inhibitor	LCt_50_ 100 mg min/m^3^ (resting inhalation) 70 mg min/m^3^ (mildly active inhalation) 15,000 mg min/m^3^ (dermal)	Days	Cholinergic crisis	Long-term neuropsychological deficits and depression	[113]

Soman	Nerve agent	Synthetic (G-series)	Rapid-ageing acetylcholinesterase inhibitor	LCt_50_ 70 mg min/m^3^ (mildly active inhalation) 10,000 mg min/m^3^ (dermal estimated)	Days	Seizures and paralysis	Severe long-term neurocognitive impairment	[113]

Sulphur mustard	Blistering agent	Synthetic	DNA alkylation leading to vesication, apoptosis and impaired repair	LD_50_ ∼0.9 g/kg (rat, oral)	Weeks–months	Blistering, blindness and airway necrosis	Carcinogenesis, infertility and pulmonary fibrosis	[122]

Tabun (GA)	Nerve agent	Synthetic (G-series)	Irreversible acetylcholinesterase inhibitor	LCt_50_ ∼400 (resting inhalation) mg min/m^3^ LD_50_ 1–1.5 mg/person (dermal)	Days–weeks	Seizures and paralysis	Central nervous system impairment and memory deficits	[113]

VX	Nerve agent	Synthetic (V-series)	Irreversible acetylcholinesterase inhibitor (lipophilic and persistent)	LCt_50_ 100 mg min/m^3^ (resting inhalation) 6–360 mg min/m^3^ (dermal)	Weeks–months	Convulsions and respiratory failure	Long-term neurological impairment	[113]

## Data Availability

All data supporting the findings of this study are available within the manuscript. The dataset is available upon reasonable request to the authors to support these findings.

## Appendix 1

  Search strategy.
  PubMed:
  ((history[Title/Abstract]) AND (chemical[Title/Abstract])) AND (warfare[Title/Abstract])
  Web of Science:
  History (Topic) and Chemical (Topic) and Warfare (Topic).
  Scopus:
  (TITLE-ABS-KEY (history) AND TITLE-ABS-KEY (chemical) AND TITLE-ABS-KEY (warfare).
